# Using the Robson classification to assess caesarean section rates in Brazil: an observational study of more than 24 million births from 2011 to 2017

**DOI:** 10.1186/s12884-021-04060-5

**Published:** 2021-08-30

**Authors:** Enny S. Paixao, Christian Bottomley, Liam Smeeth, Maria Conceicao N. da Costa, Maria Gloria Teixeira, Maria Yury Ichihara, Ligia Gabrielli, Mauricio L. Barreto, Oona M. R. Campbell

**Affiliations:** 1 Faculty of Epidemiology and Population Health, London School of Hygiene and Tropical Medicine, London, United Kingdom; 2 Centro de Integração de Dados e Conhecimentos para Saúde, Fiocruz, Salvador, Bahia Brazil; 3 Institute of Collective Health, Federal University of Bahia - Salvador (BA), Salvador, Brazil

**Keywords:** Robson classification, Caesarean section rates, Brazil, Mode of delivery

## Abstract

**Background:**

Applying the Robson classification to all births in Brazil, the objectives of our study were to estimate the rates of caesarean section delivery, assess the extent to which caesarean sections were clinically indicated, and identify variation across socioeconomic groups.

**Methods:**

We conducted a population-based study using routine records of the Live Births Information System in Brazil from January 1, 2011, to December 31, 2017. We calculated the relative size of each Robson group; the caesarean section rate; and the contribution to the overall caesarean section rate. We categorised Brazilian municipalities using the Human Development Index to explore caesarean section rates further. We estimated the time trend in caesarean section rates.

**Results:**

The rate of caesarean sections was higher in older and more educated women. Prelabour caesarean sections accounted for more than 54 % of all caesarean deliveries. Women with a previous caesarean section (Group 5) made up the largest group (21.7 %). Groups 6–9, for whom caesarean sections would be indicated in most cases, all had caesarean section rates above 82 %, as did Group 5. The caesarean section rates were higher in municipalities with a higher HDI. The general Brazilian caesarean section rate remained stable during the study period.

**Conclusions:**

Brazil is a country with one of the world’s highest caesarean section rates. This nationwide population-based study provides the evidence needed to inform efforts to improve the provision of clinically indicated caesarean sections. Our results showed that caesarean section rates were lower among lower socioeconomic groups even when clinically indicated, suggesting sub-optimal access to surgical care.

## Background

An ideal rate of caesarean sections has not been agreed, however, the progressive increase in caesarean section deliveries has intensified debate over the procedure in the last decade [1–3]. Although a caesarean section can be life-saving, it can also pose unnecessary risks to mothers and babies [4]. In the absence of a clear medical indication, the benefits of a caesarean section remain uncertain.

Identifying an unindicated caesarean section for any given woman or foetus is problematic, not least because financial incentives may encourage health systems and providers to use “soft” indications that are difficult to challenge. To address this problem, the Robson classification system has been used to group women into one of ten mutually-exclusive categories, based on six essential obstetric characteristics [5], namely: parity, previous caesarean section, gestational age, the onset of labour, foetal presentation, and number of fetuses [5]. An additional category comprises women who could not be classified because of missing or contradictory information. This instrument has been used worldwide to reduce the rates of unnecessary caesarean sections and improve obstetric care, and is indicated by WHO as a monitoring tool [6].

In Brazil, the Robson classification system has been previously applied in the national “Birth in Brazil” cohort [7], and some hospital-based studies in Sao Paulo [8] and Brasilia [9]. Using routinely collected birth registration data, the objectives of our study were to (1) estimate the caesarean rate in Brazil stratified by Robson category, (2) assess the extent to which caesarean sections were clinically indicated, and (3) identify any variation across different socioeconomic groups.

## Methods

We conducted a population-based study using routine records of the Live Births Information System (Sistema de Informação sobre Nascimentos; SINASC) in Brazil from January 1, 2011 to December 31, 2017. SINASC records all registered live births in Brazil using a standardised form that is completed by a health professional who attended the delivery. The Brazilian Ministry of Health maintains the data, and an evaluation of the birth registration system found that over 97 % of Brazilian live births were registered [10]. The form includes information on the mother (name, place of residence, age, marital status, education); the pregnancy (length of gestation, mode of delivery); and the neonate (birth weight, presence of congenital anomalies) [11]. In 2011, the form was amended to include information about the father, birth conditions, and obstetric history [12].

Because the SINASC form does not have information on previous births, we used the number of previous pregnancies as a proxy for parity. We also subdivided Robson Groups 2 and 4 into 2a and 2b and 4a and 4b to distinguish women with induced labour from those with a pre-labour caesarean section.

We estimated the rate of caesarean section stratified by maternal age (< 20, 20–24, 25–29, 30–34, 35–39, 40–44, and > 45 years), maternal education (illiterate, 1–3 years, 4–7 years, 8–11 years and more than 12 years), maternal marital status (single, widow, divorced, married), gestational age (20–21, 22–23, 24–25, 26–27, 28–29, 30–31, 32–33, 34–35, 36–37, 38–39, 40–41, > 42 weeks), birth weight (< 1,500, 1500–1999, 2000–2499, 2500–2999, 3000–3499, 3500–3999, > 4000 g), number of foetuses, delivery presentation, onset of labour, previous gestations and previous caesarean sections.

As recommended by WHO [13], we calculated the proportion of deliveries in each Robson group, the proportion of deliveries in each group that were by caesarean section, and the contribution of each group to the overall caesarean section rate (number of caesarean deliveries divided by the total number of births).

To further explore caesarean section rates within each Robson Group, we categorised Brazilian municipalities as having a very high, high, medium, or low Human Development Index (HDI), based on the 2010 Human Development Report [14], and estimated caesarean section rates stratified by HDI category.

Finally, we estimated the time trend (β coefficient which corresponds to the change in the caesarean section rate for every year) in caesarean section rates for each Robson group using either simple linear regression or the Prais-Winsten method if there was evidence of autocorrelation (Durbin-Watson statistic > 2) [15].

## Results

The Live Births Information System (SINASC) recorded 20,462,786 live births in Brazil between 2011 and 2017, 55.7 % (n = 11,405,901) of which were delivered by caesarean section. The characteristics of these births are shown in Table 1. The rate of caesarean sections was higher in older and more educated women, and increased with gestational age with a peak at 36–39 weeks (60 %). Prelabour caesarean sections accounted for more than 54 % of all caesarean deliveries.

**Table. 1:** Characteristics of 20,462,786 live births in Brazil from 2011–2017 by mode of delivery

Characteristics	Vaginal Births	Caesarean section births	Total births	Caesarean section rate (%)
**Age of the mother**				
< 20 years	2,257,865	1,507,565	3,765,430	40.0
20–24	2,651,696	2,561,203	5,212,899	49.1
25–29	2,029,296	2,934,567	4,963,863	59.1
30–34	1,334,853	2,647,268	3,982,121	66.5
35–39	619,443	1,403,459	2,022,902	69.4
40–44	152,084	330,638	482,722	68.5
≥ 45 years	11,486	21,198	32,684	64.9
Missing	162	3	165	
**Marital status of the mother**				
Single	4,473,028	4,205,309	8,678,337	48.5
Widow	15,747	22,160	37,907	58.5
Divorced	69,084	151,019	220,103	68.6
Married/union	4,377,696	6,903,721	11,281,417	61.2
Missing	121,330	123,692	245,022	
**Maternal education**				
None	101,362	36,223	137,585	26.3
1–3 years	453,747	255,239	708,986	36.0
4–7 years	2,413,955	1,712,643	4,126,598	41.5
8–12 years	5,236,732	6,339,551	11,576,283	54.8
≥ 12 years	683,137	2,879,121	3,562,258	80.8
Missing	167,952	183,124	351,076	
**Birth weight (g)**				
< 1500	119,571	151,935	271,506	56.0
1500–1999	116,372	205,613	321,985	63.9
2000–2499	504,704	630,673	1,135,377	55.5
2500–2999	2,233,728	2,465,243	4,698,971	52.5
3000–3499	3,838,057	4,666,113	8,504,170	54.9
3500–3999	1,868,805	2,609,963	4,478,768	58.3
>=4000	361,960	674,268	1,036,228	65.1
Missing	13,688	2,093	15,781	
**Number of babies**				
Singleton	8,971,608	11,046,099	20,017,707	55.2
Twins or more	73,725	346,756	420,481	82.5
Missing	11,552	13,046	24,598	
**Gestational age at delivery (weeks)**				
20–21	7,802	2,049	9,851	20.8
22–23	14,238	3,382	17,620	19.2
24–25	20,210	9,629	29,839	32.3
26–27	24,005	21,720	45,725	47.5
28–29	31,288	38,382	69,670	55.1
30–31	56,592	71,165	127,757	55.7
32–33	133,450	164,768	298,218	55.3
34–35	338,158	416,552	754,710	55.2
36–37	1,006,292	1,496,184	2,502,476	59.8
38–39	3,632,480	5,441,531	9,074,011	60.0
40–41	2,553,334	2,367,207	4,920,541	48.1
> 42	327,221	303,210	630,431	48.1
Missing	911,815	1,070,122	1,981,937	
**Delivery Year**				
2011	1,340,324	1,565,564	2,905,888	53.9
2012	1,283,546	1,615,928	2,899,474	55.7
2013	1,253,726	1,644,557	2,898,283	56.7
2014	1,277,175	1,697,954	2,975,129	57.1
2015	1,336,952	1,672,150	3,009,102	55.6
2016	1,272,867	1,583,667	2,856,534	55.4
2017	1,292,295	1,626,081	2,918,376	55.7
**Delivery presentation**				
Cephalic	8,238,500	9,562,522	17,801,022	53.7
Breech	92,719	620,941	713,660	87.0
Transverse	1,622	47,792	49,414	96.7
Missing	724,044	1,174,646	1,898,690	
**Induced labour**				
Yes	2,465,297	941,135	3,406,432	27.6
No	5,555,713	8,975,386	14,531,099	61.8
Missing	1,035,875	1,489,380	2,525,255	
**Prelabour caesarean**				
Yes	0	5,087,474	5,087,474	100.0
No	0	4,375,777	4,375,777	100.0
Not applicable (no caesarean)	7,781,452	69,172	7,850,624	0.9
Missing	1,275,433	1,873,478	3,148,911	
**Previous pregnancy and caesarean section**				
No previous pregnancy	3,033,741	4,391,353	7,425,094	59.1
Previous caesarean delivery	637,171	3,933,237	4,570,408	86.1
No previous caesarean delivery	4,130,500	1,856,212	5,986,712	31.0
Missing	1,225,099	1,255,473	2,480,572	
**HID**				
Very High	1,667,342	2,402,466	4,069,808	59.0
High	4,503,476	6,801,017	11,304,493	60.2
Medium	2,599,014	2,120,271	4,719,285	44.9
Low	287,009	82,147	369,156	22.3

*NA* not applicable

*For women with a caesarean, these are not applicable for other, poorly defined reasons

A total of 15,426,356 (75.4 %) records had information on all six core variables required for the Robson classification. Women with a previous caesarean section was the largest group (Group 5, 21.7 %), followed by primigravidae women with a single cephalic pregnancy at 37 + weeks gestation in spontaneous labour (Group 1, 16.1 %) and primigravidae women who had labour induced or had a prelabour caesarean section (Group 2, 17.2 %) (Table 2).

**Table. 2:** Relative size and caesarean delivery rates in Brazil using 10- group classification, 2011–2017

Robson Group	Group definition	Proportion of pregnancies	Caesarean section rate	Contribution of caesarean section to overall deliveries	WHO recommended caesarean rates
		**%**	**%**	**%**	**%**
1	Nulliparous women with a single cephalic pregnancy, ≥ 37 weeks gestation in spontaneous labour	16.1	43.8	7.1	< 10 %
2	Nulliparous women with a single cephalic pregnancy, ≥ 37 weeks gestation who had labour induced or were delivered by CS before labour	17.2	67.6	11.6	20–35 %
2a	Labour induced	7.32	24	1.8	-
2b	Pre-labour CS	9.86	100	9.9	-
3	Multiparous women without a previous uterine scar, with a single cephalic pregnancy, > 37 weeks gestation in spontaneous labour	18.6	17.5	3.3	< 3 %
4	Multiparous women without a previous CS, with a single cephalic pregnancy, ≥ 37 weeks gestation who had labour induced or were delivered by CS before labour	11.2	42.3	4.7	15 %
4a	Labour induced	7.21	10.3	0.7	-
4b	Pre-labour CS	4.01	100	4	-
5	All multiparous women with at least one previous CS, with a single cephalic pregnancy, ≥ 37 weeks gestation	21.7	84.9	18.4	50–60 %
6	All nulliparous women with a single breech pregnancy	1.38	89.4	1.2	-
7	All multiparous women with a single breech pregnancy including women with previous CS	1.86	84.4	1.6	-
8	All women with multiple pregnancies including women with previous CS	2	82	1.7	60 %
9	All women with a single pregnancy with a transverse or oblique lie, including women with previous CS(s)	0.22	96.8	0.2	-
10	All women with a single cephalic pregnancy < 37 weeks gestation, including women with previous CS	9.7	48.8	4.8	30 %

Groups 6–9 (breeches, multiple pregnancies, and transverse/oblique lies), for whom caesarean sections would be indicated in most cases, all had caesarean section rates above 80 %, as did women who had had a previous caesarean (Group 5). Primigravid women at term, singleton, and cephalic pregnancy in spontaneous labour (Group1) were almost twice as likely to deliver by caesarean section as those with induced labour (Group 2a) (43.8 % vs. 24.0 %). Women with a previous caesarean section and primigravid women accounted for two-thirds of all caesarean sections, a third each. Notably, prelabour caesarean section of primigravid women or multigravida without previous section (2b and 4b) accounted for 25.3 % of all caesarean sections ( Table 2).

**Fig. 1:**
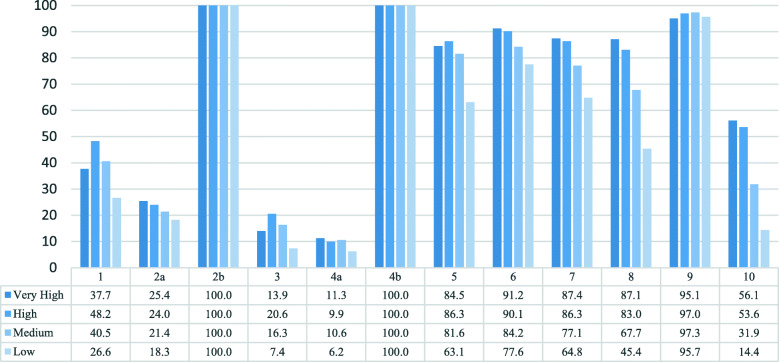
Caesarean delivery rates in Brazil using 10- group classification, stratified by HDI group, 2011–2017

In general, women who live in municipalities with a higher HDI were more likely to deliver by caesarean section; the most considerable difference in the caesarean section rates between very high and low categories was observed in groups 8 and 10 (Fig. 1). This trend was apparent across all Robson groups, except for group 9 where rates of caesarean section were uniformly close to 100 % irrespective of HDI group.

The general Brazilian caesarean section rate remained stable during the study period (β = 0.33; p = 0.178). Analyses of time-trends by Robson group showed that the caesarean section rate increased in groups 5 and 8 (β = 0.87; p < 0.01; β = 0.05; p = 0.016 respectively) and decreased in groups 2, 4 9 and 10 (β=-0.73; p < 0.01; β=-0.27; p < 0.01; β=-0.02; p = 0.006; β=-0.17; p = 0.013). In the remaining groups, the rates did not change significantly over the years.

A group of 5,036,430 (24.6 %) women could not be classified because of missing or contradictory data, However, the percentage of missing data dropped annually, from 34 % to 2011 to 8.3 % in 2017). We identified 95,472 inconsistent records, most of them (91,333) reported live births or stillbirth/abortion and no previous pregnancy.

## Discussion

In Brazil, caesarean section rates remained stable in the period from 2011 to 2017, always showing very high levels by international standards. The actions implemented in the country to stimulate vaginal delivery could have been effective in containing this increase, but not in reducing it [16]. Our analyses of Robson groups show stable temporal trends and patterns that can be used to characterise practice and suggest improvement.

Robson groups can be separated into those where caesarean section might be absolutely or strongly indicated (Groups 6 to 10) and those with a less strong indication (Groups 1–4). The caesarean section rates in every Robson group are higher than suggested by the WHO Robson guideline, except in group 9 where the recommendation and practice is 100 %. Even groups with seemingly favourable conditions for vaginal delivery, such as women with a singleton, term, cephalic pregnancy, and no previous caesarean (1–4), had an average caesarean rate of higher than 40 %.

For Group 5 (previous caesarean section), rates of 50–60 % would be appropriate, according to WHO standards, however, we see a rate of 84.9 %. Because of high historical rates of caesarean section, nearly a quarter of births are to women who are in Group 5. Given the rarity of vaginal birth after caesarean section (VBAC) this group will continue to drive high levels into the future. In groups 6, 7 and 9 the rates are 89.4, 84.4 and 96.8 % respectively, which is appropriate given guidelines on caesarean section for abnormal lie. In Group 8, one should usually expect a rate of around 60 % but we see 82 %. For Group 10, usually around 30 %, we see 48.8 %.

In terms of the relative distribution of the Brazilian obstetric population within Robson groups, the results were similar to those in previous literature. Primigravid women with a single cephalic pregnancy accounted for 1/3 of the obstetric population, and similar findings have been found in France (38.2 %) [17], Canada (39.7 %) [18] and Sri Lanka (38.1 %) [13]. However, prelabour caesarean in Groups 2b and 4b accounted for 25 % of the overall caesarean rate, a meaningfully higher rate than observed in other settings, such as the USA [19] and Peru [20] where this group accounts for less than 10 %. The Robson classification does not include information on indication for caesarean section. Therefore, it is not possible to know if the prelabour caesarean section occurred due to medical indication. However, it is plausible to speculate that in part, these groups had a caesarean delivery for reasons of medical or maternal preference.

Women in spontaneous labour (Group 1 and 3) were more likely to deliver by caesarean section than those with induced labour (Group 2a 4a). One explanation for these findings could be that some of the women in Groups 1 and 3 may have been misclassified and could potentially belong to another group with a higher risk of caesarean delivery or maybe some of them had an intervention carried out without any clear medical indication. This is because maternal request or the physician’s preference have consistently been identified as a cause for growing rates of caesarean sections in recent decades [21–23] and this is a common practice in Brazil [24].

Similar findings were previously found for the group of patients with a previous caesarean section in countries such as the USA [19] and in the previous study conducted in Brazil [7]. There is a common misconception in Brazil that after a caesarean section, the following delivery must be a caesarean section, especially in the private sector, where repeated caesarean sections can be higher than 97 %. Previous studies have shown that in the public sector, these rates can be much lower among eligible women (52.2 %), and our data showed that in municipalities with lower HDI, it can be around 65 % [25]. However, there is a lack of studies on the effects of the mode of delivery among women with a previous uterine scar. Although some studies have shown that an anterior caesarean section seems to be an important risk factor for the occurrence of placental accretism in later pregnancy, which leads to severe postpartum hemorrhage and maternal death from this cause [26], the evidence available in the literature is controversial. Maternal morbidity and mortality were lower following VBAC compared with repeated caesareans in a study performed in China [27] but the opposite was seen among Canadian women [28].

Group 10, premature births, accounted for more than 8 % of caesarean section, slightly higher than that observed in the USA[19]. The rate of caesarean section in this group was also higher than observed in other countries, such as the USA [19], Palestine [29] and Sri Lanka [13].

However, these results were not homogenous in the entire country. There was a notable difference in caesarean rates with varying local socioeconomic conditions. This is demonstrated in the municipalities with the lowest HDI index where there were lower caesarean section rates than in wealthier places. Similar results have been seen in the Robson classification for a multicountry dataset to explore caesarean section trends by HDI [30]. This is even seen for abnormal lie where the recommendations for caesarean section are strong, suggesting women in more deprived areas may be getting less appropriate care.

### Strengths

To our knowledge, this study is the most extensive investigation into caesarean section deliveries in Brazil. The results of this study were similar to the large cohort “Birth in Brazil” however, our study used live birth records (administrative data) of all births in Brazil and “Birth in Brazil” used interviews with a random large sample of women. This is an indication of the quality of the data used in the present study. The use of HDI enables the monitoring of the rate of caesareans and early recognition of any changes in rates of caesareans on a smaller and more homogeneous scale.

### Limitations

The main limitation is the lack of data for some of the core variables used to classify women into one of the Robson groups. The proportion of missing data occurred disproportionately by mode of delivery; it was more frequent among women who delivered by caesarean section, therefore this is a potential source of bias. However, the proportion of missing data decreased over time, suggesting the data quality has been improving since the form was enhanced (2011). SINASC-Brazil was previously shown to underreport information on some core variables needed to classify women into one of the Robson categories, such as gestational age. There is also evidence that the quality of the information has great spatial heterogeneity [31]. Another limitation is that the caesarean section rates in Group 9 were lower than 100 %, as recommended by WHO [5], and this could indicate either poor data quality or poor quality of care.

## Conclusions

Brazil is a country with one of the world’s highest caesarean section rates. This nationwide population-based study provides the evidence needed to take steps towards improving the provision of clinically indicated caesarean sections. Our results suggest that among higher socioeconomic groups, a caesarean section is undertaken for a high proportion of women with a clinical indication, however, they are also commonly carried out without a clear clinical indication. Among lower socioeconomic groups, caesarean section rates were lower even when clinically indicated, suggesting sub-optimal access to surgical care.

## Data Availability

The data that support the findings of this study are available on request from the CIDACS/ FIOCRUZ, and ethical approval. The data are not publicly available due to restrictions as they contain information that could compromise the privacy of research participants.

## Abbreviations

SINASC: Live Birth Information System
CIDACS: Centre for Data and Knowledge Integration for Health
HDI: Human Development Index
